# Improving Access to Anti-Schistosome Treatment and Care in Nonendemic Areas of China: Lessons from One Case of Advanced Schistosomiasis Japonica

**DOI:** 10.1371/journal.pntd.0001960

**Published:** 2013-01-17

**Authors:** Da-Bing Lu, Li Zhou, Ying Li

**Affiliations:** 1 Department of Epidemiology and Statistics, School of Public Health, Soochow University, Suzhou, China; 2 Anhui Institute of Parasitic Diseases, Hefei, China; 3 School of Biology and Basic Medical Sciences, Soochow University, Suzhou, China; National Institute of Parasitic Diseases China CDC, China

## Introduction

**Figure 1:**
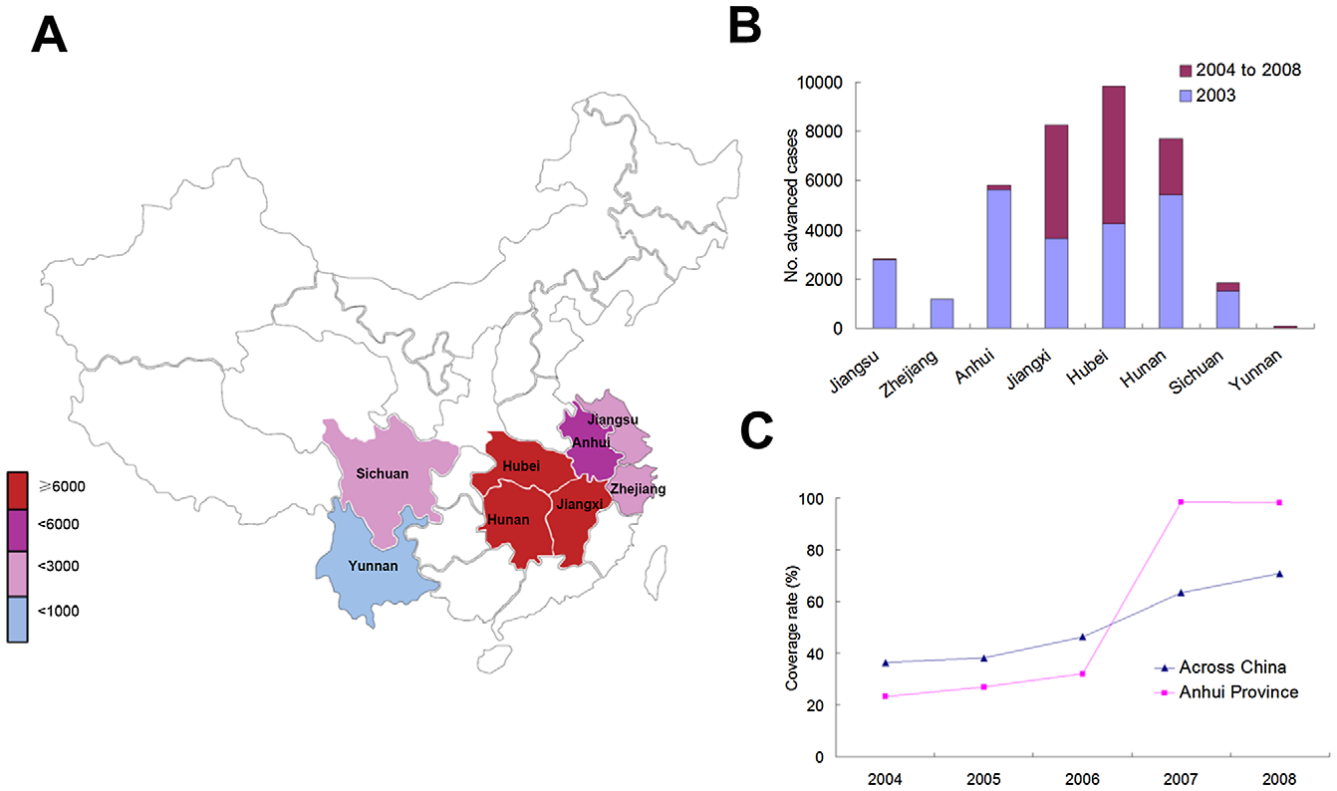
Numbers of advanced schistosomiasis japonica in China. (A) In each province by 2008; (B) in each province by 2003 and new cases found from 2004 to 2008; and (C) the coverage rates of the aid-project in the cases at the levels of nation or province (Anhui) over 2004 to 2008.

Schistosomiasis, caused by trematode blood flukes of the genus *Schistosoma*, is the second most prevalent disease after malaria in the tropical and subtropical world. Among five schistosome species, *Schistosoma japonicum* is the most serious, mainly because of its high egg output [1]. This parasite is mainly endemic in mainland China, the Philippines, and Indonesia. Advanced schistosomiasis, due to either an infection without chemotherapy or repeated infections over time, is the most serious form of schistosomiasis and is usually characterized by periportal liver fibrosis, spleen enlargement and congestion, portal hypertension, and other serious consequences [2]. The suffering for an advanced patient caused by schistosomes could last for a lifetime, as the associated pathological changes on the host, if not properly treated, could develop toward a more serious condition. In addition, *S. japonicum* in humans has been estimated to be able to live for an average of 4.5 years [3]. Prolonged survival of the parasite to as long as about 47 years has been seen in an American who emigrated from the Philippines and continued to show active eggs in rectal biopsies [4]. These eggs can continue to produce damages to organs where they are deposited. Therefore, the treatment of the disease usually includes both anti-schistosome treatment and symptomatic therapy. The former is based on fecal examinations and serological tests, which are usually negative in most cases [5], or endoscopic examination. The etiological treatment is comparatively easier when without any serious complication, and therefore, much more attention and financial aids are going to the latter. In the Zhejiang province of China for example, in 2003 there remained 1,187 advanced cases who still needed medical assistance, although the disease there had been interrupted a decade ago [6]. Such a looming problem has been suggested to exist for perhaps as long as another half century in China [7]. Fortunately, with the economic development and well-improved standards of living over the last decades in China, in 2004 the government of China redefined schistosomiasis control, together with the control of HIV/AIDS and tuberculosis, as one of its highest priorities in communicable disease control [8]. As one main part of the integrated control measures, the project for treatment of advanced schistosomiasis has been initiated across the endemic regions of China. Box 1 lists a series of regulations on that aspect made by the government. The main point of this is that a screening system for advanced schistosomiasis among local residents will be set up in each endemic county and, if a farmer is diagnosed with advanced schistosomiasis (see Box 2 for the diagnosis criteria), the involved medical expense will be mostly covered by different levels of government. Figure 1 shows the numbers of advanced cases and newly found cases in each province and, as the consequence of the project, the increasingly rising coverage rate of advanced cases [9]. The direct or long-term estimated benefit from this project, as estimated by the work [10], would be quite considerable. Here we presented a case observed in a nonendemic area within the Anhui Province of China to point out the demerit of the ongoing project and raise some aspects yet to improve. This follow-up investigation was approved by an ethics committee in both the Anhui Institute of Parasitic Diseases and Soochow University. A consent form was obtained from the patient, and she gave consent to have her case details published.

**Box 1. The aid-project for treatment of advanced schistosomiasis in China since 2004**

1. The notice of further consolidation on schistosomiasis control (The State Council of China, 2004).

An advanced schistosomiasis patient is provided with appropriate financial assistance from both central and local governments for the incurred medical care.

2. The surgical treatment-aid project for advanced schistosomiasis among farmers (The Ministry of Health of China, 2005).

A total of RMB5000 (US$605 in 2004) for the incurred operation is provided from the central government, and the rest of the cost is provided by local governments.

3. The nonsurgical treatment-aid project for advanced schistiosomiasis among farmers (The Ministry of Health of China, 2006).

A total of RMB3000 (US$375 in 2006) for one common patient or a maximum of RMB6000 (US$750 in 2006) for the serious is provided by the central government and the rest of the cost by local governments.

4. According to the above requirements and also depending on local economic status, provinces each drew up their regulations. For example, in Anhui, after the regulations had been performed for 2 years, the aid level has been adjusted to RMB2000 (US$263 in 2007) per farmer per year since 2007 (Public Health Department of Anhui, 2007).

**Box 2. The criteria for the diagnosis of advanced schistosomiasis (The Ministry of Health of China, 2006)**

1. Water contact history.

2. Symptoms or physical signs of portal hypertension, or of colon granuloma or dwarfism.

3. Eggs or miracidia from faeces, or eggs in colon under an endoscopic examination.

## The Special Case

A 75-year-old woman was admitted to Anhui Provincial hospital (in Hefei city of Anhui) for investigation of stomach diseases on April 24, 2010. She grew up in Taizhou city, one area previously endemic with the parasite, of Jiangsu Province, and since the 1960s had moved to Hefei city, a nonendemic area. In the past she had occasionally visited her hometown, but each time she stayed there for no more than 1 week. She had never been to any other endemic areas. The patient reported repeated epigastric discomfort, anorexia, and eructation for about 20 days. On admission her systolic/diastolic blood pressure was measured as 176/102 mm Hg, and other physical and neurological examinations showed no abnormalities. The haematological and biochemical laboratory findings were as follows: white blood cell count (WBC) 3,500 per µl with 75.9% neutrophilic granulocyte; erythrocyte count 4.07×10^6^ per µl; haemoglobin 120.0 g/L; platelet count 83,000 per µl; glucose 7.10 mmol/L; and the others were within normal ranges. Tests for hepatitis A, B, C, and E were negative. A routine chest radiograph proved normal. Endoscopic examination revealed diffuse inflammation in the rectum and sigmoid colon. Ultrasonography of the abdomen showed diffuse pathological changes of liver (characteristic fish-scale pattern caused by schistosomiasis) and seroperitoneum. Computed tomographic angiography of the superior mesenteric artery showed splenomegaly and a dilated portal vein (an indication of portal hypertension). The patient was released from the hospital on May 5, 2010 after nearly 2 weeks of anti-symptomatic treatment and was recommended to go to a parasitic special hospital for the management of the possible advanced schistosomiasis. The patient then on the same day went to the Anhui Institute of Parasitic Diseases in Hefei city of Anhui, whose responsibility it is to organize and inspect the treatment and control of the disease across the whole province. There she was given advice on the choice of a few candidate hospitals or schistosomiasis control stations located in endemic areas within the province, which are highly experienced in the diagnosis and treatment of the disease.

On May 7, 2010, the patient went to Xiuning Schistosomiasis Control Station located in Xiuning county, 323 km away from Hefei. She was given both a serological test (the Indirect Haemagglutination Assay) for *S. japonicum* antibodies and an ultrasonography of the abdomen. The serologic test for schistosomes was negative. The result from the ultrasonography was the same as that reported in the previous hospital but suggested a possible development of the hepatic cirrhosis. However, the patient did not receive any treatment from the medical facility.

**Figure 2:**
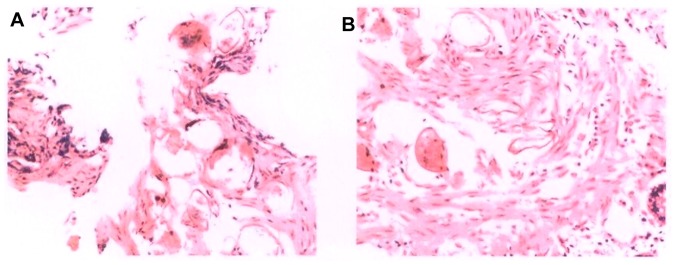
*S. japonicum* egg nodes visible in the submucosa. (A) The ascending colon and (B) the sigmoid colon.

Then the patient, on May 8, 2010, went to and was admitted by the Hefei Second Hospital (in Hefei city of Anhui) with primary diagnoses of a combination of schistosomal hepatic fibrosis, schistosomal enteropathy, erosive gastritis, and electrolyte disturbances. A series of routine examinations and tests, as the above, were given on admission. The results from endoscopic examination first observed egg nodes within the submucous section of colon (see Figure 2). After nearly 1 month of anti-symptomatic treatment, the patient, with a slight improvement in abdominal pain and anorexia, was discharged from the hospital on June 3, 2010. Once more, she was recommended to go to a hospital that specialized in schistosomiasis for further anti-parasite treatment.

With the help of a senior health worker from the Anhui Institute of Parasitic Diseases, the patient was admitted to the Tongling Schistosomiasis Control Station in Tongling county (172 km away from Hefei) on June 4, 2010. Both serological and stool examination for the existence of *S. japonicum* were negative, and the ultrasonograph showed liver fibrosis at an early stage and an enlarged spleen. Combined with the results of the endoscopic examination, the patient was then diagnosed with advanced schistosomiasis with megalospleny and erosive gastritis. Following that, the patient was treated with praziquantel 60 mg/kg for 3 days. During the course of treatment, the patient reported significant epigastric discomfort and dizziness, and therefore, the course of treatment was not completed. On June 10, 2010, the patient was allowed to leave the hospital and was advised to go to a high-level hospital for further treatment.

## Lessons

**Figure 3:**
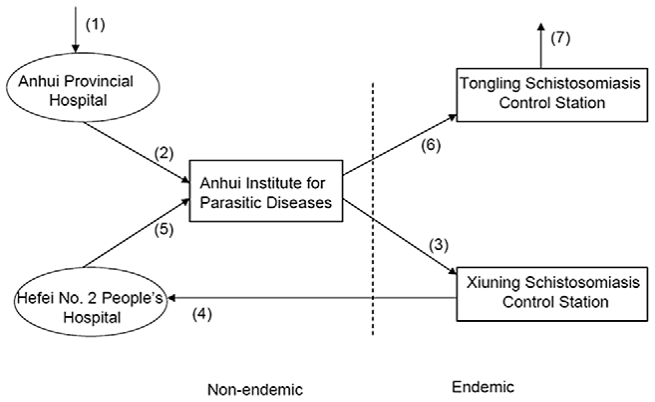
The diagram of the path in pursing medical treatment for the patient.

During a period of one and a half months, the patient went through two hospitals, one preventive institute, and two regional schistosomiasis control stations (Figure 3), three of which she had been admitted into for medical treatments. The Anhui Provincial Hospital, where the patient first received a medical examination and treatment, is one of the best and top hospitals within the province, with advanced equipment and technology and well-educated staff. Given the results of her liver and spleen ultrasonography and the fact that she originally came from an endemic area but had no history of treatment for schistosomiasis, the patient was initially diagnosed with chronic schistosomiasis and recommended for further examination. In the Hefei No. 2 Hospital, *S. japonicum* egg nodes were found within the submucous section in her colon through endoscopic examination. Although the patient was not misdiagnosed from the beginning, she did go through five medical or health service facilities in pursuing medical treatment, covering a distance of nearly 1,000 km. As effective praziquantel treatment can, to some extent, reverse hepatic fibrosis and associated changes at the early stage [11], the patient, without any anti-schistosome treatment in her history, may be in an urgent need for killing possible parasites alive or eggs active within her body. More importantly, as the patient currently lives in a nonendemic area and does not know where she should/could go for the appropriate treatment, she indeed needs more aid from the government in both guidance of medical care and any possible financial assistance.

The case we reported here reflected the flaw of the ongoing aid project. According to the document made by the Ministry of Health of China, a system for registration and follow-up investigation of suspected advanced schistosomiasis patients among local residents is created in each of the endemic or previously endemic counties or cities. A resident with splenomegaly or hepatic changes caused by schistosomes is registered with their local schistosomiasis control station and reported to higher health authorities. The suspected person is then subjected to a thorough inquiry of medical history, a health check-up, a set of laboratory examinations, and ultrasonography by an expert group. When he or she is diagnosed with advanced schistosomiasis, he or she will be enrolled into the treatment-aid project. Following that, a detailed treatment plan will be made depending on the health status of the patient. Figure 1 reveals that quite a number of new advanced cases had been found from 2004 to 2008; particularly in both of the two provinces of Hubei and Jiangxi, the numbers had nearly doubled in 5 years. This partially indicates the efficiency of the system in local areas. This system was set in currently or previously endemic areas and targets the local residents only, on which the aid project was based. In nonendemic areas, however, there is no special administrative facility or office sharing the duty of monitoring and reporting such patients among local residents. Therefore, the number of advanced cases that occurred in such areas—not to mention providing the treatment aid supported by government to these patients—remains unknown. Due to an unprecedented migration rate of populations in China as well as in other developing countries [12], the number of such missed or ignored advanced cases could be huge. It is no doubt that the disability estimates of over 70 million DALY (disability-adjusted life-years) [13] could have still been underestimated. Therefore, an improvement in the current monitoring system and aid-project management (i.e., enrolling vulnerable populations who have migrated from an endemic to nonendemic areas) (see Box 3) is clearly and urgently needed. This seems of great importance when the control and elimination of schistosomiasis has been put on the agenda [14].

**Box 3. The lessons**

1. The ongoing aid-project should be extended to the patients in nonendemic areas.

2. A facility or authority must be clarified for the implementation of the project in such areas described above.

3. The quality of diagnosis and treatment for schistosome infection should be strengthened in all local anti-schistosome stations.

In previous work [15], out of 109 advanced cases, nearly 31% (*n* = 34) presented with erosive gastritis. The patient reported here also concurrently suffered from erosive gastritis and hypertension, which could have resulted in the failure in the anti-schistosomal praziquantel treatment. It is hypothesized that the pathological change in a liver, due to the deposit of schistosome eggs, leads to portal hypertension. This may hamper the blood circulation in surrounding vascular systems and then result in congestion and edema in gastric mucosa. This may predispose the patient to the infections of *Helicobacter pylori* or other bacteria, and thus, erosive gastritis may develop.

We also noticed that the patient, after being to a schistosomiasis control station, went to the Hefei No. 2 People's Hospital for nearly 1 month of hospital treatment, where flexible colonoscopy revealed nodular lesions in the colon caused by eggs. This finally confirmed the diagnosis of advanced disease. However, such a key approach for identification of advanced cases has been neglected in most local stations since the 1990s. It therefore seemed necessary for a schistosomiasis control station, a key unit in local disease control, to be able to conduct the job.
